# *STAT*3 polymorphism and *Helicobacter pylori* CagA strains with higher number of EPIYA-C segments independently increase the risk of gastric cancer

**DOI:** 10.1186/s12885-015-1533-1

**Published:** 2015-07-19

**Authors:** Gifone A Rocha, Andreia MC Rocha, Adriana D Gomes, César LL Faria, Fabrício F Melo, Sérgio A Batista, Viviane C Fernandes, Nathálie BF Almeida, Kádima N Teixeira, Kátia S Brito, Dulciene Maria Magalhães Queiroz

**Affiliations:** Laboratory of Research in Bacteriology, Faculdade de Medicina, Universidade Federal de Minas Gerais, Av. Alfredo Balena, 190 s/216, 30130-100 Belo Horizonte, Brazil

**Keywords:** Gastric cancer, *STAT*3 gene polymorphism, *STAT*3 rs744166, *Helicobacter pylori*, CagA, EPIYA-C segments

## Abstract

**Background:**

Because to date there is no available study on *STAT*3 polymorphism and gastric cancer in Western populations and taking into account that *Helicobacter pylori* CagA EPIYA-C segment deregulates SHP-2/ERK-JAK/STAT3 pathways, we evaluated whether the two variables are independently associated with gastric cancer.

**Methods:**

We included 1048 subjects: *H. pylori*-positive patients with gastric carcinoma (*n* = 232) and with gastritis (*n* = 275) and 541 blood donors. Data were analyzed using logistic regression model.

**Results:**

The rs744166 polymorphic G allele (*p* = 0.01; OR = 1.76; 95 % CI = 1.44-2.70), and CagA-positive (OR = 12.80; 95 % CI = 5.58-19.86) status were independently associated with gastric cancer in comparison with blood donors. The rs744166 polymorphism (*p* = 0.001; OR = 1.64; 95 % CI = 1.16-2.31) and infection with *H. pylori* CagA-positive strains possessing higher number of EPIYA-C segments (*p* = 0.001; OR = 2.28; 95 % CI = 1.41-3.68) were independently associated with gastric cancer in comparison with gastritis. The association was stronger when host and bacterium genotypes were combined (*p* < 0.001; OR = 3.01; 95 % CI = 2.29-3.98). When stimulated with LPS (lipopolysaccharide) or Pam3Cys, peripheral mononuclear cells of healthy carriers of the rs744166 GG and AG genotypes expressed higher levels of *STAT*3 mRNA than those carrying AA genotype (*p* = 0.04 for both). The nuclear expression of phosphorylated p-STAT3 protein was significantly higher in the antral gastric tissue of carriers of rs744166 GG genotype than in carriers of AG and AA genotypes.

**Conclusions:**

Our study provides evidence that *STAT*3 rs744166 G allele and infection with CagA-positive *H. pylori* with higher number of EPIYA-C segments are independent risk factors for gastric cancer. The odds ratio of having gastric cancer was greater when bacterium and host high risk genotypes were combined.

## Background

Gastric cancer is the third leading cause of cancer-related death in the world and one of the most common malignancies [1] especially in developing countries where the prevalence of *Helicobacter pylori* remains high.

*H. pylori* infection leads to a persistent chronic gastric inflammation by inducing the expression of inflammatory cytokines that play an important role in the development of gastric cancer.

Polymorphisms in genes encoding pro-inflammatory cytokines have shown to increase the risk of gastric cancer, specifically interleukin-1β (*IL1B*-511/31, interleukin-1 receptor antagonist (IL1RN*2*)*, and tumour necrosis factor A (*TNFA*-308) [2–6]. However, gastric cancer is a multifactorial disease and several genetic factors might be involved in the pathogenesis of the tumour.

There are evidences that signal transducer and activator of transcription protein 3 (STAT3) is implicated in the development and progression of cancer and plays a role in inducing neoplastic transformation. STAT3 participates in a series of tumourigenic processes including cell proliferation, cell survival, anti-apoptosis, angiogenesis, immune evasion and inflammation [7]. STAT3 is constitutively activated in several human cancers including skin, head and neck, ovarian, breast, colon, prostate and gastric cancer [8–14]. The mechanism of STAT3 activation relies on the stimulation by interleukin-6 (IL-6) cytokine family, in particular IL-6 and IL-11 and JAK (janus kinase)/STAT3-SHP (tyrosine phosphatase)-2/ERK (extracellular-signal-regulated kinases)1/2 signalling pathways emanating from the signal transducer glycoprotein (gp)130 [15, 16]. SHP-2 is a feedback inhibitor of JAK/STAT3 signalling. Indeed, higher expression of SHP-2 by gain-of-function in mutant mouse results in reduced phosphorylation of STAT3 [17]. Otherwise, increased STAT3 secretion is observed in knock-in mice harbouring mutation abrogating SHP-2/ERK1/2. Notably, these knock-in mice develop gastric tumour [18–20].

The SNP (single nucleotide polymorphism) rs744166 in the intron 2 of *STAT*3, located on chromosome 17q21, has recently been associated with gastric cancer in a Chinese population [21], as well as colorectal cancer in American population of European origin [22] and non-small-cell lung cancer in a Chinese population [23]. Associations between other *STAT*3 polymorphisms and solid tumours have also been demonstrated [24, 25].

In addition to host factors, *H. pylori* virulent factors increase the risk of gastric cancer, notably the cytotoxin-associated antigen (CagA) encoded by the *cag*A gene (cytotoxin-associated gene A). Patients infected with CagA-positive *H. pylori* strains either possessing EPIYA-D or higher number of EPIYA-C segments are at increased risk of gastric cancer [6, 26–30]. CagA is injected into gastric epithelial cells, via bacterial type-IV secretion system, where it undergoes tyrosine phosphorylation at specific carboxi-terminal region comprising a variable number of EPIYA (Glu-Pro-Ile-Tyr-Ala) segments. Four distinct EPIYA segments designated EPIYA-A, B, C and D have been described according to different amino acids flanking each EPIYA motif. *H. pylori* strains circulating in Western countries possess EPIYA-A and EPIYA-B that are followed by 0-3 EPIYA-C segments, whereas *H. pylori* strains from East Asian countries possess EPIYA-A, EPIYA-B and EPIYA-D sites. Phosphorylated EPIYA-C or EPIYA-D motif acts as a specific binding site that interacts with SH2 domain-containing SHP-2 abnormally triggering the SHP-2/mitogen-activated protein kinases (MAPK)/ERK1/2-JAK/STAT3 pathways. It is worth mentioning that CagA possessing EPIYA-D or higher number of EPIYA-C segments binds more robustly to SHP-2 [31].

Taking these considerations together and the fact that to date there are no studies evaluating the *STAT*3 polymorphism and risk of gastric cancer in Western populations; we assessed the *STAT*3 polymorphism and CagA status as well as EPIYA-C pattern of *H. pylori* strains and risk of gastric cancer.

Therefore, the data were analyzed in logistic regression models in order to identify variables independently associated with increased risk of gastric cancer. We also evaluated whether the polymorphism might be functional by assessing the ability of peripheral blood mononuclear cells (PBMCs) from healthy volunteer carriers and non carriers of the polymorphic allele to express STAT3 mRNA. Finally, we evaluated the nuclear expression of phosphorylated (p)-STAT3 protein in the gastric mucosa of gastritis patients according to the *STAT*3 genotypes.

## Methods

This study was approved by the Ethics Committees of Universidade Federal de Minas Gerais (UFMG ETIC 018/00) and Comissão Nacional de Ética (CONEP 096/02) and written informed consent was obtained from all subjects.

In total, 1048 subjects were included: 275 *H. pylori*-positive patients with chronic gastritis, 232 *H. pylori*-positive patients with distal gastric carcinoma and 541 voluntary healthy blood donors (68.4 % *H. pylori*-positive). The patients were selected among those who underwent endoscopy for the evaluation of symptoms related to the upper gastrointestinal tract or underwent gastric surgery to remove gastric carcinoma at University Hospital/UFMG and Luxemburgo Hospital in Belo Horizonte, Brazil. The blood donors were from Fundação Hemominas, Minas Gerais, Brazil.

Most of the included individuals (>80 %) were of low socioeconomic level with similar cultural habits and all were native of Minas Gerais state with the same ethnic background, approximately 33 % of Portuguese, 33 % of Amerindians and 33 % of African ancestry, homogeneously present in each individual [32].

In the gastritis patients, endoscopic biopsy samples of the antral and oxyntic gastric mucosa were obtained for histological and microbiological studies (culture, urease test and PCR for *H. pylori* specific genes) to determine *H. pylori* status. Antral and oxyntic biopsy specimens were routinely processed and histological sections were stained with carbolfuchsin [6] for *H. pylori* investigation and hematoxyllin and eosin for histological evaluation according to the updated Sydney System [33]. Mononuclear (MN) and polymorphonuclear (PMN) cell infiltrations were graded as absent (0), mild (1), moderate (2), or marked (3). In the gastric cancer patients, the fragments for the analyses of the presence of *H. pylori* were obtained from the stomach removed by gastrectomy within one hour of resection. The patients were also submitted to the ^13^C-urea breath test imediatly before endoscopy to determine *H. pylori* status. The patients were considered to be *H. pylori* positive when the culture was positive or when two among the other tests were positive.

### Amplification of the *ure*A and *cag*A genes

All isolates were tested for the presence of specific *H. pylori ureA* gene [34]. The set of primers used and the reaction conditions are described in Table 1. The standard Tx30a *H. pylori* strain was used as a positive control, and an *Escherichia coli* strain and distilled water were both used as negative controls.

**Table 1:** Primer pairs and conditions used in the PCR to detect *H. pylori genes* (*ureA*, *cagA* and *3’ variable region of the* cag*A* gene that contains EPIYA sequences) and in qRT-PCR to genotype *STAT*3 rs744166 and to evaluate *STAT*3 mRNA expression

Gene	Primers (5’ – 3’)	PCR conditions	PCR Product (bp)	Reference
*ureA*	F: GCCAATGGTAAATTAGTT	95 °C - 5 min.; 34 cycles (94 °C 1 min., 45 °C - 1 min. and 72 °C - 1 min.) and 72 °C - 5 min.	411	34
	R: CTCCTTAATTGTTTTTAC			
*cag*A	F:CTGCAAAAGATTGTTTGCGAGA	95 °C - 5 min, 34 cycles (95 °C 1 min, 50 °C-1 min and 72 °C-1 min) and 72 °C - 15 min	400	35
	R:AGACGGTTTGTTAGAAAACGTC			
*cag*A	F:GATAACAGGCAAGCTTTTGAGG	95 °C - 5 min, 38 cycles (94 °C 1 min, 55 °C - 1 min and 72 °C - 2 min.) and 72 °C - 7 min.	349	36
	R:CTGCAAAAGATTGTTTGCGAGA			
*cagA* 3’ variable region	F: ACCCTAGTCGGTAATGGGTTA	95 °C - 5 min, 35 cycles (95 °C 1 min, 50 °C-1 min and 72 °C-1 min.) and 72 °C - 7 min	500 - 850	37
	R: GTAATTGTCTAGTTTCGC			
*STAT*3^a^ Rs744166	CTGTTTGTTCTATAAATTACTGTCA[A/G]GCTCGATTCCCTCAAGACATTACAG	60 °C - 1 min., 95 °C - 10 min., 50 cycles (95 °C - 15 s., 50 °C - 90 s.) and 60 °C - 1 min.	70	

Bp, base pairs; ^a^, Applied Biosystem, HS00374280-m1

In the patients, the *cag*A gene was amplified by means of two previously described primer pairs [35, 36]. A *H. pylori* strain from our collection (1010-95), known to be *cag*A-positive, was used as a positive control, and Tx30a *H. pylori* strain lacking *cag*A and distilled water were both used as negative controls. The primers used and the reaction conditions are described in Table 1. The *H. pylori* strains were considered to be *cag*A-positive when at least one of the two reactions was positive.

### Amplification of the *cag*A EPIYA region

For the PCR amplification of the 3’ variable region of the *cag*A gene that contains the EPIYA sequences, 20 to 100 ng of DNA were added to 1 % Taq DNA polymerase buffer solution (KCl 50 mM and Tris-HCl 10 mM), 1.5 mM MgCl_2_, 100 μM of each deoxynucleotide, 1.0 U Platinum Taq DNA polymerase (Invitrogen, São Paulo, Brazil), and 10 pmol of each primer, for a total solution volume of 20 μL. The primers used were previously described by Yamaoka et al. [37] and are listed in Table 1. The amplified products were electrophoresed in 1.5 % agarose gel that was stained with ethidium bromide, and analyzed in an ultraviolet light transilluminator. The reaction yielded products of 500 to 850 bp according to the number of EPIYA-C segments.

To confirm the results, the 3’ variable region of the *cag*A gene was sequenced. Briefly PCR products were purified with the Wizard SV Gel and PCR Clean-Up System (Promega, Madison, MI) according to the manufacturer’s recommendations. Purified products were sequenced using a BigDye® Terminator v3.1 Cycle Sequencing Kit in an ABI 3130 Genetic Analyzer (Applied Biosystems, Foster City, CA). The sequences obtained were aligned using the CAP3 Sequence Assembly Program (available from: http://pbil.univ-lyon1.fr/cap3.php). After alignment, nucleotide sequences were transformed into amino acid sequences using the Blastx program (available from: http://blast.ncbi.nlm.nih.gov/Blast.cgi) and compared to sequences deposited into the GenBank (http://www.ncbi.nlm.nih.gov/Genbank/).

### *H. pylori* and CagA Status in the blood donors

In the blood donors, *H. pylori* status was investigated by using a commercial ELISA kit (Cobas Core anti-*H. pylori*, EIA Roche, Basel, Switzerland), which was previously validated for the Brazilian population being 95.4 % sensitive and 100 % specific [38]. CagA status was investigated by serology using a commercial ELISA kit (Helicobacter pylori p120 CagA; Viva Diagnostika, Hürth, Germany), which was also previously validated for the Brazilian population being 97.4 % sensitive and 88.9 % specific [39].

### *STAT3* SNP rs744166 polymorphism

The *STAT*3 single nucleotide polymorphism (SNP) rs744166 was chosen to be investigated because in genome wide studies the G allele was shown to be a protector factor for inflammatory bowel disease [26], which might be attributed to a higher production of STAT3, well known as protector of intestinal mucosa, but associated with gastric cancer in animal models [18–20].

The SNP rs744166 in the intron 2 of *STAT3*, located on chromosome 17q21, was analyzed in DNA extracted from leukocytes with the QIAamp DNA mini kit (QIAgen) by pre-designed Taqman SNP genotyping assay on ABI7500 real time PCR system (Applied Biosystems, Foster City, CA). The primer used and the reaction conditions are described in Table 1.

### *in vitro* stimulation of *STAT*3

In order to evaluate the effect of the *STAT*3 rs744166 different genotypes on the mRNA expression, we used PBMCs from nine healthy subjects (three AA, three AG and three GG) of the laboratory team who were *H.* pylori negative, as determined by ^13^C-urea breath test. Blood samples were obtained from each subject after 8-h fast in five different days and were independently assayed. PBMCs were freshly isolated using Ficoll-Paque Plus (Amersham Biosciences, GE Healthcare, São Paulo, Brazil) and 9 x 10^5^ cells were seeded in triplicate in 24 well plates containing RPMI 1640 supplemented with 10 % fetal bovine serum, 100 U/mL penicillin and 100 μg/mL streptomycin in 5 % CO_2_, 95 % humidity at 37 °C for 24 h. Thereafter, PBMCs were stimulated with LPS, an agonist of toll-like-receptor-4 (TLR-4), 100 ng/mL from *Escherichia coli* 055:B5 (Sigma, St Louis, MO) or Pam3Cys, an agonist of TLR-2, 100 ng/mL (EMC Microcollections GmbH, Tuebingen, Germany) both diluted in 500 μL RPMI 1640 and incubated in 5 % CO_2_, 95 % humidity at 37 °C for 6 h. Unstimulated cells were used as a negative control. According to an initial protocol time course experiment, six hours proved to be an ideal time point to detect *STAT3* expression after stimulation with both LPS and Pam3Cys.

### *STAT*3 RNA extraction

Total RNA was purified by using RNeasy Mini Kit (QIAgen). The extracted RNA was then treated with RQ1 RNase-free DNase (Promega, São Paulo, Brazil). The RNA concentration was determined by spectrophotometry using NanoDrop 2000 (Thermo Scientific, Wilmington, NC). One hundred nanograms of RNA were reverse transcribed to cDNA using a High Capacity cDNA (Applied Biosystem). A cDNA synthesis reaction including all components except the reverse transcriptase was subsequntly used as control for quantitative real time PCR (qRT-PCR).

### Relative quantification of the *STAT*3 transcripts

The *STAT*3 calibrator was purified cDNA isolated from PBMCs containing *STAT*3 sequence. The remaining calibrators were made by a serial two-fold dilution (20.0 ng/μL - 1,280.0 ng/μL) and tested in triplicate to determine the most effective PCR amplification conditions. The calibration curve produced r^2^ value of 0.99. The *STAT*3 mRNA expression was analyzed by using Taqman primers and probe (Applied Biosystems). The qRT-PCR was performed with thermal cycling conditions of 50 °C-2 min, 95 °C-10 min, 50 cycles of 95 °C-15 s and, 60 °C-1.5 min. Distilled water was also used as a negative control.

Relative quantification of the *STAT*3 transcripts was determined by means of algebraic calculation using the method 2^-∆∆Ct^ [40], normalized to the glyceraldheyde 3-phosphate dehydrogenase.

### Immunohistochemistry

Because it is known that expression of STAT3 in tumour tissue depends on the stage of the tumour [41, 42], the p-STAT3 nuclear expression was assessed in formalin-fixed paraffin-embedded sections of the antral and corpus mucosa of gastritis patients harbouring rs744166 AA, AG and GG genotypes, eight in each group, who were randomly selected, by conventional immunohistochemistry. To retrieve antigenicity, the sections were placed in 10 mmol/L citrate buffer solution, pH 6.0, and heated in a microwave for 12 min. Samples were then treated with 3 % hydrogen peroxide-metanol for 12 min to block endogenous peroxidase and, rinsed with distilled water. The assay was performed using as primary antibody 1:50 diluted rabbit anti-human phospho-STAT3 (pTyr-705) IgG (Sigma, Cambridge, UK) with modifications including incubation with Novocastra Post Primary Block for 30 min and with NovoLink polymer for 30 min (Novocastra Laboratories Ltd, São Paulo, Brazil). Sections were counterstained with Meyer’s hematoxycillin and then mounted. Negative controls were carried out by omission of the primary antibody. Known immunostaining positive slides were used as positive controls. The gastric epithelial cells with the nucleus stained in brown were considered positive for p-STAT3. The number of p-STAT3-positive cells (proportional to the number of all cells) was evaluated in 20 representative visual fields at a magnification of 400X in an Olympus CX41RF microscope. Slides were examined by two independent observers who were blinded to the other results. The percentage of positive cells for p-STAT3 was classified as 0 (none), 1 (≤50 %), 2 (50 to 90 %), and 3 (> 90 %) and staining intensity was graded in three step scale, as follows: 1 (low), 2 (medium) and 3 (high) intensity.

### Statistical analysis

The associations of the *STAT*3 genotypes with the number of EPIYA-C segments and gender were evaluated by the *χ*^2^ test with Yates’ correction and the mean age by the Student’s *t* test or ANOVA and scores by the Kruskal-Wallis test. Regarding to *STAT*3 mRNA expression, scores of nuclear p-STAT3 in the gastric cells and the degree of gastric inflammation, the differences between the groups were evaluated by the two-tailed Mann-Whitney *U*-test. *p* values ≤ 0.05 were considered as significant. The associations of the variables including the rs744166 *STAT*3 genotypes (treated as variables having 3 values with 0 meaning the AA genotype, 1 the carrier of one G allele and 2 the GG genotype), CagA status, CagA EPIYA-C patterns, age and gender were investigated in logistic regression models by comparing gastric cancer patients with blood donors or gastritis patients (dependent variable). Association between the polymorphism and *H. pylori* status was also evaluated in a logistic regression model in the group of blood donors. Finally, in other model, we investigated whether the combined score of the number of EPIYA-C segments and *STAT*3 polymorphism increased the risk for gastric cancer. All variables with p values ≤ 0.25 were included in the multivariate analysis. Odds ratio (OR) and 95 % confidence interval (CI) were used as an estimate of the risk. The Hosmer-Lemeshow goodness-of-fit test was used to evaluate the fit of the model [43].

Data were analyzed with SPSS, version 17 (SPSS Inc., Chicago, IL).

## Results

### Demographic data and rs744166 *STAT*3 polymorphism

The characteristics of the population are listed in Tables 2 and 3.

**Table 2:** Distribution of the *STAT*3 rs744166 genotypes according to the gender, mean age, *H. pylori* status and CagA status in blood donors (*n* = 541)

	Gender		*STAT*3 genotypes			
	Female	Male	AA	AG	GG	Total
	n (%)	n (%)	n (%)	n (%)	n (%)	n (%)
Female	132 (24.4)	-	39 (29.5)	63 (47.8)	30 (22.7)	132 (100)
Male	-	409 (75.6)	142 (34.7)	187 (45.7)	80 (19.6)	409 (100)
total	-	-	181 (33.5)	250 (46.2)	110 (20.3)	541 (100)
Mean age in yrs (SD)	33.6 (9.9)	33.8 (9.6)	34.6 (10.3)	33.6 (9.8)	32.9 (9.8)	33.8 (10.0)
Range yrs	18 - 59	18 - 65	18 - 65	18 - 64	19 - 59	18 - 65
Hp ^**-ve**^	53 (40.2)	118 (28.9)	53 (29.3)	81 (47.4)	37 (21.6)	171 (31.6)
Hp ^**+ve**^	79 (59.8)	291 (71.1)	128 (34.6)	169 (45.7)	73 (19.7)	370 (68.4)
CagA ^**-ve**^	34 (43.0)	126 (43.3)	51 (31.9)	77 (48.1)	32 (20.0)	160 (43.3)
CagA ^**+ve**^	45 (57.0)	165 (56.7)	73 (34.8)	95 (45.2)	42 (20.0)	210 (56.7)

n, number; SD, standard deviation; yrs, years; Hp, *Helicobacter pylori*; ^-ve^, negative; ^+ve^, positive

**Table 3:** Distribution of the *STAT*3 rs744166 genotypes according to the gender, mean age, CagA status and EPIYA-C patterns in patients with gastritis and gastric cancer

Disease	*STAT*3 genotypes			
	AA	AG	GG	Total
	n (%)	n (%)	n (%)	n (%)
Gastritis (*n* = 275)				
Female	61 (33.7)	78 (43.1)	42 (23.2)	181 (65.8)
Male	27 (28.7)	36 (38.3)	31 (33.0)	94 (34.2)
Mean age in yrs (SD)	49.6 (17.1)	53.9 (18.6)	58.6 (15.3)	53.8 (17.6)
*cag*A ^**-ve**^	26 (25.7)	49 (48.5)	26 (25.7)	101 (36.7)
*cag*A ^**+ve**^	62 (35.6)	65 (37.4)	47 (27.0)	174 (62.3)
EPIYA-AB	1 (25.5)	1 (25.5)	2 (50.0)	4 (2.3)
EPIYA-ABC	44(32.1)	53 (38.7)	40 (29.2)	137 (78.7)
EPIYA-ABCC	15 (53.6)	8 (28.6)	5 (17.8)	28 (16.1)
EPIYA-ABCCC	2 (40.0)	3 (60.0)	0	5 (2.9)
Gastric cancer (*n* = 232)				
Female	19 (21.8)	41 (47.1)	27 (31.0)	87 (37.5)
Male	40 (27.6)	61 (42.1)	44 (30.3)	145 (62.5)
Mean age in yrs (SD)	63.4 (13.3)	61.5 (12.9)	61.7 (13.6)	62.0 (13.2)
*cag*A ^**-ve**^	2 (10.5)	11 (57.9)	6 (31.6)	19 (8.2)
*cag*A ^**+ve**^	57 (26.8)	91 (42.7)	65 (30.5)	213 (91.8)
EPIYA- AB	0	2 (66.7)	1 (33.3)	3 (1.4)
EPIYA-ABC	28 (23.1)	53 (43.8)	40 (33.1)	121 (56.8)
EPIYA-ABCC	22 (30.1)	30 (41.1)	21 (28.8)	73 (34.3)
EPIYA-ABCCC	7 (43.8)	6 (37.5)	3 (18.7)	16 (7.5)

n, number; SD, standard deviation; yrs, years. ^-ve^, negative; ^+ve^, positive

In the blood donors (Table 2), *H. pylori* infection was more frequently observed in males than in females (*p* = 0.02; OR = 1.65; 95 % CI = 1.08 – 2.54), but, when the data were adjusted for the socio-economical level, the association disappeared (*p* < 0.001; OR = 0.53; 95 % CI = 0.40 – 0.70 for socio-economical level and p = 0.12 for gender). The CagA positivity (*p* = 0.93; OR = 0.99; 95 % CI = 0.58 – 1.68) and the mean age (*p* = 0.85) did not differ when males and females were compared (Table 2). Also, the frequency of *STAT*3 AA, AG and GG genotypes did not differ between males and females (*χ*^2^ = 1.38, 2 degrees of freedom, *p* = 0.50) and between *H. pylori*-positive and -negative status (*χ*^2^ = 0.73, 2 degrees of freedom, *p* = 0.69).

In the group of gastritis, the distribution of *STAT*3 genotypes was not different in respect to the gender (*χ*^2^ = 3.05, 2 degrees of freedom *p* = 0.22) and *cag*A status (*χ*^2^ = 3.91, 2 degrees of freedom, p = 0.14), but a tendency of association was observed between the distribution of EPIYA-C segments and *STAT*3 genotypes (*χ*^2^ = 4.73, 2 degrees of freedom, p = 0.09), the infection by strains with EPIYA-CC or EPIYA-CCC being less frequent in the GG genotype carriers (Table 3). In respect to the EPIYA pattern, neither difference in the mean age (*p* = 0.65) nor in the gender (*χ*^2^ = 0.25, 2 degrees of freedom, *p* = 0.88) was observed (Table 4).

**Table 4:** Distribution of the EPIYA-C patterns according to the gender and mean age, in patients with gastritis and gastric cancer

Disease	EPIYA pattern			
	AB	ABC	ABCC	ABCCC
	n (%)	n (%)	n (%)	n (%)
Gastritis (*n* = 275)				
Female	2 (1.7)	93 (80.2)	18 (18.1)	3 (2.6)
Male	2 (3.4)	44 (75.9)	10 (19.5)	2 (3.5)
Mean age in yrs (SD)	44.3 (25.0)	54.7(16.9)	50.6 (15.1)	52.0 (11.0)
Range	27 - 73	18 - 86	27 - 73	39 - 63
Cancer (*n* = 232)				
Female	1 (1.2)	47 (56.7)	29 (42.1)	6 (7.2)
Male	2 (1.5)	74 (56.9)	44 (41.6)	10 (7.7)
Mean age in yrs (SD)	56.5 (3.5)	60.2 (14.4)	64.4 (13.2)	70.0 (9.7)
Range	54 - 59	28 - 89	33 - 92	55 - 85

n, number; SD, standard deviation; yrs, years

In the gastric cancer patients, there was no significant difference in the distribution of the *STAT*3 genotypes and the number of EPIYA-C segments (*p* = 0.16; Kruskal Wallis test) and *cag*A status (*χ*^2^ = 2.73, 2 degrees of freedom, *p* = 0.26) (Table 3). The gender neither associated with the *STAT*3 genotypes (*χ*^2^ = 1.03, 2 degrees of freedom *p* = 0.60) nor with the number of EPIYA-C segments (*χ*^2^ = 0.03, 2 degrees of freedom, *p* = 0.98), (Table 4). Infection by strains with higher number of EPIYA-C segments increased with increasing age (*p* = 0.004).

### Comparisons between the groups

No significant difference was observed between the group of gastritis patients and blood donors regarding to the *STAT*3 genotype distribution (*p* = 0.75; OR = 1.05; 95 % CI = 0.76-1.45) and CagA status (*p* = 0.22; OR = 1.19; 95 % CI = 0.76-1.45).

By comparing the blood donors with the patients with gastric cancer (Table 2 and Table 3), a significant difference was observed in the frequency of CagA positive status (*p* < 0.001; OR = 8.54; 95 % CI = 4.99 – 14.76) and in the distribution of the *STAT*3 genotypes (*χ*^2^ = 10.86, 2 degrees of freedom, *p* = 0.004). By stratifying the gender, in respect to the *STAT*3 genotypes, the association remained for male gender (*χ*^2^ = 7.54, 2 degrees of freedom p = 0.02), but not for female gender (*χ*^2^ = 2.57, 2 degrees of freedom, *p* = 0.28).

When the gastritis patients were compared with the gastric cancer patients, in the carriers of the rs744166 AA genotype, the frequency of infection with CagA strains with one EPIYA-C segment was significantly higher in the former (*p* = 0.01; OR = 2.68; 95 % CI = 1.17 – 6.28). Otherwise, in the carriers of AG (*p* = 0.002; OR = 3.27; 95 % CI = 1.42 – 7.67) or GG (*p* = 0.01; OR = 4.2; 95 % CI = 1.32 – 14.20) genotypes, infection with strains with two or three EPIYA-C segments were more frequently observed in the gastric cancer than in the gastritis patients (AB was not included in the analysis and ABCC and ABCCC segments were analysed together in all analyses) (Table 3).

Infection by strains with higher numbers of EPIYA-C segments was more frequently observed in the gastric cancer than in the gastritis patients: females (*p* < 0.001; OR = 3.49; 95 % CI = 1.75 – 6.97) and males (*p* = 0.007; OR 2.68; 95 % CI = 1.23 – 5.93) (Table 4).

The number of EPIYA-C segments was positively associated with the mean age of the patients with gastric cancer (*p* = 0.004), but not with the gender (*p* = 0.99) (Table 4). In the group with gastritis, the number of EPIYA-C segments neither associated with mean age nor with gender (*p* > 0.64) (Table 4).

### Association between *STAT*3 rs744166 genotypes and gastric cancer

The alleles were in Hardy-Weinberg equilibrium in the controls (p = 0.19).

In the blood donors, a logistic regression demonstrated that the *STAT*3 genotypes were not associated with *H. pylori* infection*,* even including the age and gender in the model (*p* = 0.66; OR = 0.94; 95 % CI = 0.73 – 1.22).

Compared with blood donors, the rs744166 polymorphic G allele, CagA-positive status, age and gender were independently associated with gastric cancer (Table 5).

**Table 5:** Host and bacterium variables associated with gastric cancer (*n* = 232) in comparison with *H. pylori*-positive blood donors (*n* = 370)

Covariate	Univariate analysis	Multivariate analysis		
	*p*	OR	95 % CI	*p*
Age	< 0.001	1.20	1.16 - 1.23	< 0.001
Gender	< 0.001	2.99	1.48 - 6	0.002
rs744166	0.002	1.76	1.14 - 2.70	0.01
CagA ^**+ve**^	< 0.001	12.80	5.48 - 29.86	< 0.001

The Hosmer-Lemeshow test showed good fit (10 steps; 8 degrees of freedom; *p* = 0.20). rs744166 genotype was treated as categorical variables having 3 values with 0 meaning the AA genotype, 1 the carrier of one G allele and 2 the GG genotype, ^+ve^, positive

Next, we compared gastric carcinoma patients with those with gastritis in order to evaluate the number of EPIYA-C segments as a predictor of gastric cancer as well as the co-participation of the bacterial EPIYA-C segments and *STAT*3 polymorphism in the risk of the disease. The logistic regression analysis showed that the rs744166 polymorphism and higher number of EPIYA-C segments were independently associated with gastric cancer (Table 6). Harbouring *STAT*3 polymorphism plus infection with CagA strains possessing higher number of EPIYA-C segments was more strongly associated with gastric cancer (Table 6).

**Table 6:** Host and bacterium variables associated with gastric cancer (*n* = 213) in comparison with gastritis (*n* = 174) in patients infected by *H. pylori cag*A-positive strains

Covariates	Univariate analysis	Multivariate analysis		
	*p*	OR	95 % CI	*p*
First model^a^				
Age	< 0.001	1.04	1.02 - 1.05	< 0.001
Gender	< 0.001	3.58	2.10 - 6.09	< 0.001
rs744166	0.007	1.64	1.16 - 2.31	0.005
EPIYA-C number	< 0.001	2.28	1.41 - 3.68	0.001
Second model^b^				
Age	< 0.001	1.05	1.05 - 1.06	< 0.001
Gender	< 0.001	3.23	2.02 - 5.17	< 0.001
rs744166 plus EPIYA-C number	< 0.001	3.01	2.29 - 3.97	< 0.001

The Hosmer-Lemeshow tests showed good fit for models ^a^(10 steps, 8 degrees of freedom; *p* = 0.42) ^b^(10 steps, 8 degrees of freedom; *p* = 0.25), OR, odds ratio; 95 % CI: confidence interval. We constructed two models studying the loci, according to the amount of G rs744166 allele, treated as categorical variables having 3 values with 0 meaning the AA genotype, 1 the carrier of one G allele and 2 the GG genotype. In the second model the variable was obtained by the sum of the amount of *STAT*3 G allele and the number of EPIYA-C segments

### *STAT*3 rs744166 polymorphism and microscopic aspects of the gastric mucosa

Results of previous studies have shown that infection with *H. pylori* CagA-positive strains are associated with more prominent gastric inflammation and infection with CagA strains possessing higher number of EPIYA-C segments are associated with pre-malignant gastric lesions. Thus to avoid interference of those variables, in the group of patients with gastritis infected with CagA-negative strains we analyzed the association between *STAT*3 and gastritis by treating rs744166 genotypes as dichotomous variables: AA = 0 and AG + GG = 1. The presence of G allele was associated with higher degree of mononuclear cells in the corpus mucosa (median = 1, range, 0-1 vs. median = 2, range, 1-3; *p* = 0.01).

### Association between rs744166 polymorphism and *in vitro* expression of *STAT*3

**Fig. 1:**
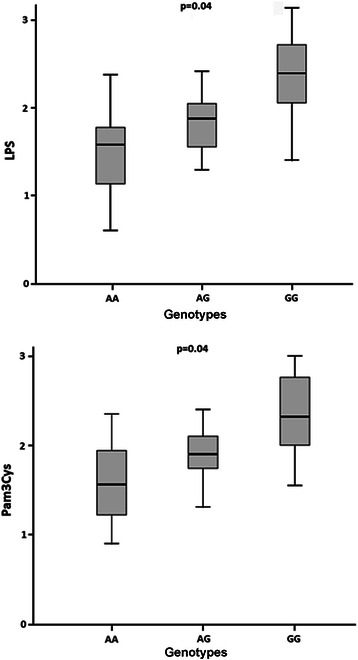
Box plots representing mRNA STAT3 expression of five independent measurements, performed in triplicate. The upper and lower limits of the boxes represent the 75th and 25th percentiles, respectively; the horizontal bar across the box indicates the median and the end of the vertical lines indicate the minimum and maximum data values. Quantitative real-time PCR was used to analyze the influence of *STAT*3 rs744166 genotypes on the STAT3 mRNA expression before and after LPS or Pam3Cys stimulation. Gene expression was normalized to the expression of a reference gene, glyceraldehyde 3-phosphate dehydrogenase (GAPDH). PBMCs of healthy *H. pylori*-negative subjects, as determined by ^13^C-urea breath test, carrying rs744166 GG (*n* = 3), AG (n = 3) or AA genotypes (*n* = 3) were stimulated with 100 μg/mL LPS or 100 μg/mL Pam_3_Cys for 6 h. RNA was purified and *STAT*3 mRNA expression analyzed by qRT-PCR. A cDNA synthesis reaction including all components except the reverse transcriptase was subsequently used as control for quantitative real time PCR (qRT-PCR). Distilled water was also used as a negative control

When stimulated with LPS or Pam3Cys, PBMCs of the healthy carriers of the GG genotype expressed significantly higher amounts of *STAT*3 mRNA than carriers of the AG or AA genotype (Fig. 1).

### Expression of p-STAT3 protein according to the *STAT*3 genotypes

**Fig. 2:**
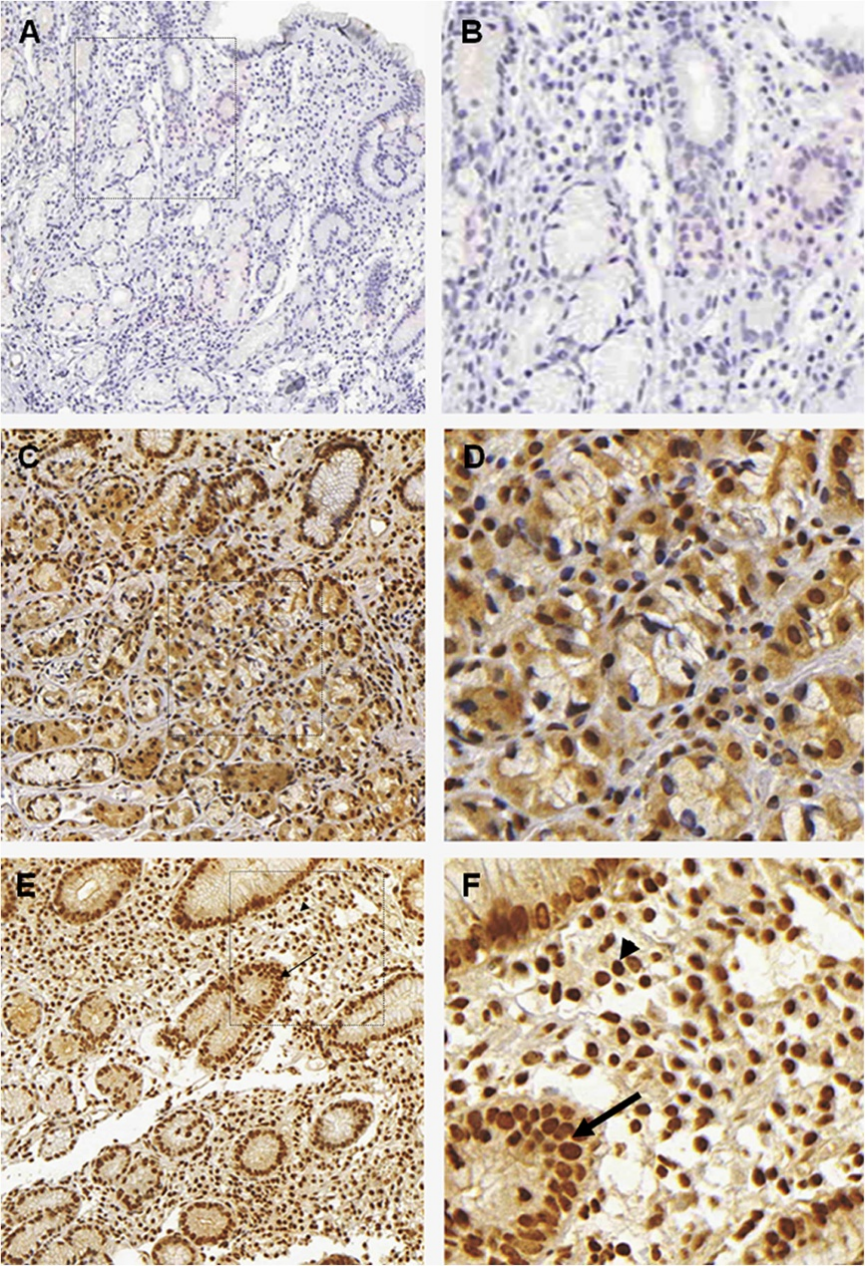
Representative immunohistochemical staining showing nuclear localization of p-STAT3 in the antral glandular cells and stroma cells under low-power (100x) and high-power (400x) magnification of carriers of AA and GG *STAT*3 rs744166 genotypes: **a** and **b**, mock; **c** and **d**, medium brown-colored nuclei in the gastric stroma cells and glandular cells of a carrier of AA genotype and **e** and **f**, densely stained nuclei of 100 % of cells, both of the gastric stroma (arrow head) and glands (arrow) in a carrier of GG genotype. The percentage of positive cells for STAT3 was classified as 0 (none), 1 (≤ 50 %), 2 (50 to 90 %), and 3 (> 90 %) and staining intensity was graded in three step scale, as follows: 1 (low), 2 (medium) and 3 (high) intensity

**Fig. 3:**
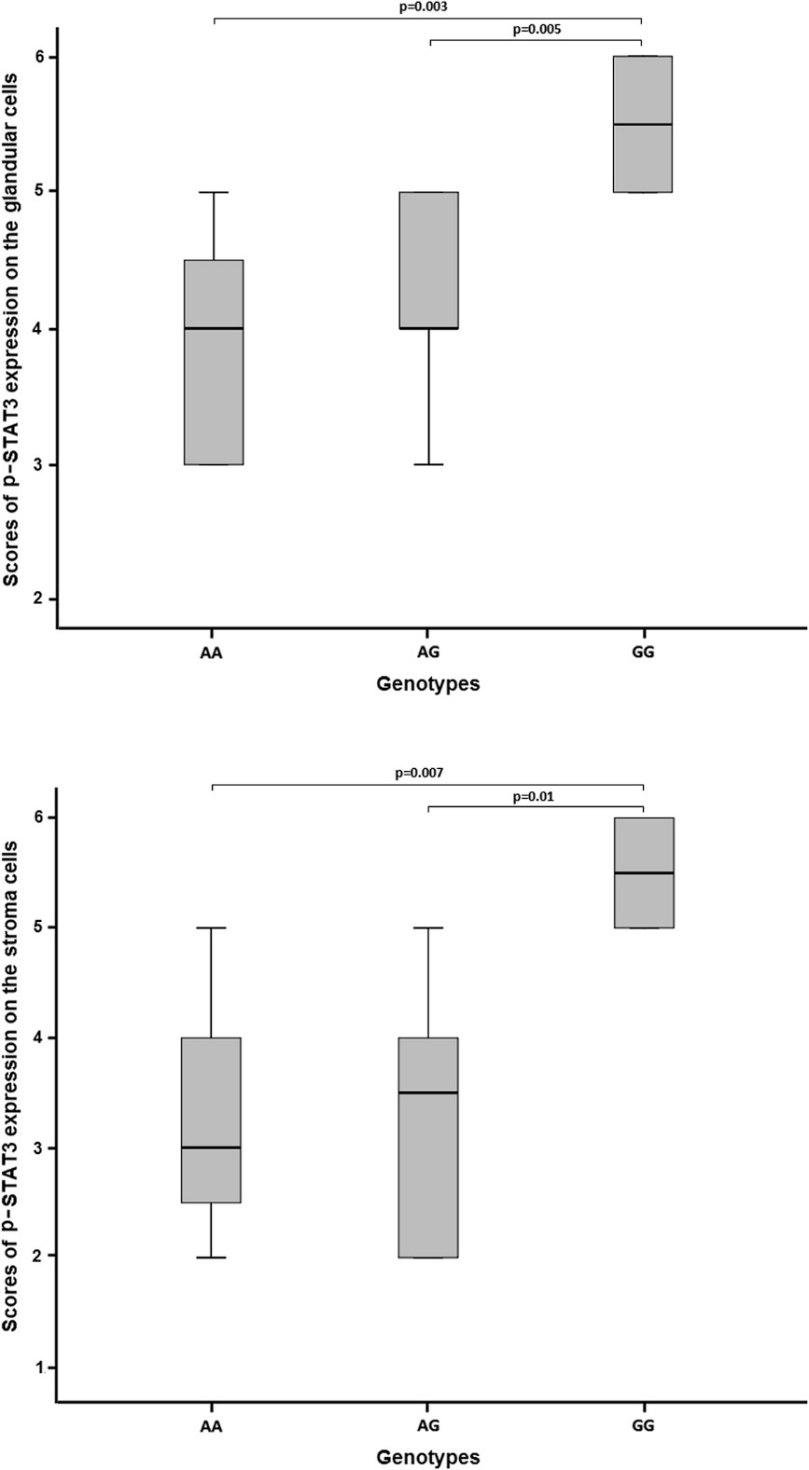
Box plots representing scores of nuclear p-STAT3 expression in the antral glandular cells and stroma cells of patients with gastritis according to the different *STAT*3 rs744166 gentotypes. The upper and lower limits of the boxes represent the 75th and 25th percentiles, respectively; the horizontal bar across the box indicates the median and the end of the vertical lines indicate the minimum and maximum data values

Figure 2 shows nuclear localization of p-STAT3 in the antral glandular cells and stroma cells of carriers of AA and GG *STAT*3 rs744166 genotypes. The percentage of p-STAT3 expression was significantly higher in the nuclei of the antral glandular cells (*p* = 0.005 and *p* = 0.003) and in the gastric stroma cells (*p* = 0.01 and *p* = 0.007) of the patients carrying the rs744166 GG genotype than in those carrying AG or AA genotype, respectively, but no differences were observed between the genotypes AA and AG (*p* ≥ 0.49) (Fig. 3).

## Discussion

To the best of our knowledge, this is the first study to demonstrate that *STAT*3 rs744166 G allele is associated with increased risk of gastric cancer in a Western population. Conversely, the results of the study in a Chinese population [21] showed that G allele was associated with a decreased risk of gastric cancer, which may be explained by differences in the role of host polymorphisms and risk of gastric cancer between Western and Eastern populations including polymorphism in genes coding *IL1B*-511 and IL1RN*2 [1–6, 44].

There are growing evidences suggesting that STAT3 has a crucial role in carcinogenesis. STAT3 regulates expression of several genes involved in a variety of cellular responses including proliferation, differentiation, apoptosis and wound healing [45]. STAT3 activity supports tumour-cell survival by up regulating expression of the anti-apoptotic protein BCL-X_L_ (B-cell lymphoma-2-like) and many other proteins involved in cell proliferation and survival including myeloid cell leukaemia 1 (MCL1), cyclin D1 and MYCC [14, 46]. Furthermore, STAT3 selectively induces and maintains a pro-inflammatory microenvironment that further supports tumour progression.

The results of the present study are in opposite to that observed in patients with Crohn’s disease who are more frequently carriers of the A than the G allele [26]. There are evidences that physiologic consequences of the multiple STAT3 actions depend on the specific cell types involved [45]. STAT3 plays a protective role in the intestinal mucosa as demonstrated in knockout mice with intestinal epithelial cell-specific deletion of STAT3 that exhibit more severe induced acute colitis than the control group with a functioning STAT3 [19, 47]. It has been suggested that increased STAT3 signalling may predispose to up regulation of trefoil factor family3 (*TFF*3) gene expression in intestinal globet cells and protection against IBD [19] and downregulates trefoil factor family-1 (*TFF*1), a gastric-specific tumour suppressor, predisposing to neoplastic transformation [48]. In fact, mice with knock-in mutation located at Y757F on gp130, which destroys the docking site for SOCS3 that negatively regulates STAT3 activation, develop antral gastric cancer [19, 20, 49]. In addition, in the present study we observed that *H. pylori*-positive individuals harbouring AG or GG genotypes had high degree of mononuclear cells in the gastric corpus, considered a pre malignant lesion.

Although to date, no functional role has been attributed to *STAT*3 rs744166 polymorphism, in this study, the expression of *STAT*3 mRNA was significantly higher in stimulated PBMCs from a group of healthy volunteers, carrying the GG genotype, than from those carrying AG or AA genotype. Supporting these findings we also found that the phosphorylated protein STAT3 expression was significantly higher in the gastric tissue of patients carrying the GG genotype than in those carrying AG or GG genotype. Based on these findings, we might suppose that *STAT*3 rs744166 polymorphism is functional, or is in Hardy-Weinberg disequilibrium with other polymorphism that increases *STAT*3 transcription and STAT3 secretion.

Of note, in this study we observed differences in the risk of gastric cancer according to the gender; when gastric cancer patients were compared with blood donors, the G allele being associated with increased risk of gastric cancer only in males. Otherwise, although infection with higher number of EPIYA-C segments was associated with pre malignant lesions (data not shown) and gastric cancer in both males and females, we found that the odds ratio of having gastric cancer was more robust in females (3.49 vs. 2.69). Based on these findings, one may speculate that there are different pathways of carcinogenesis between males and females. EPIYA-C binds to one or two SH2 domains of SHP-2, which in turn potentiates Ras-ERK signalling pathway that results in oncogenic stress [50], meanwhile, *STAT*3 polymorphism possibly activates JAK/STAT3 pathway. However, we can not rule out that the differences observed might be due to a bias because the number of females is smaller than that of males in the blood donors.

When we compared gastritis and gastric cancer patients, we also demonstrated that the association was more robust in the presence of concurrent *STAT*3 polymorphism and the infection with CagA strains with higher number of EPIYA-C segments, highlighting the relevance of both bacterial and host factors as predictors of gastric cancer. Remarkably we found that in patients with gastric cancer the rs744166 GG genotype and infection with CagA strains with higher number EPIYA-C segments predominate.

## Conclusions

In conclusion, the present study shows that *STAT*3 rs744166 polymorphism and infection with *H. pylori* with CagA possessing higher number of EPIYA-C segments are independent risk factors for gastric cancer in the evaluated population. Therefore, individuals with genetic polymorphisms associated to *STAT3* who are colonized by CagA strains with higher number of EPIYA-C segments are most likely to benefit from *H. pylori* eradication aiming to prevent gastric cancer as it has been recommended by the IARC working group on *Helicobacter pylori* eradication as a strategy for preventing gastric cancer. According to the group all countries should explore the possibility of introducing population-based *H. pylori* screening and treatment programmes especially for subpopulations that appear most likely to benefit from interventions.
